# Multiplier method may be unreliable to predict the timing of temporary hemiepiphysiodesis for coronal angular deformity

**DOI:** 10.1186/s13018-017-0604-1

**Published:** 2017-07-10

**Authors:** Zhenkai Wu, Jing Ding, Dahang Zhao, Li Zhao, Hai Li, Jianlin Liu

**Affiliations:** 1 Department of Pediatric Orthopaedics, Xin-Hua Hospital affiliated to Shanghai Jiao Tong University School of Medicine, No. 1665, Kongjiang Road, Shanghai, 200092 China; 2 Department of Pediatric Orthopaedics, Ying-Hua Medical Group of Bone and Joint Healthcare in Children, No. 358, Haibo Road, Shanghai, 200000 China

**Keywords:** Temporary hemiepiphysiodesis, Multiplier method, Coronal angular deformity

## Abstract

**Background and purposes:**

The multiplier method was introduced by Paley to calculate the timing for temporary hemiepiphysiodesis. However, this method has not been verified in terms of clinical outcome measure. We aimed to (1) predict the rate of angular correction per year (ACPY) at the various corresponding ages by means of multiplier method and verify the reliability based on the data from the published studies and (2) screen out risk factors for deviation of prediction.

**Methods:**

A comprehensive search was performed in the following electronic databases: Cochrane, PubMed, and EMBASE™. A total of 22 studies met the inclusion criteria. If the actual value of ACPY from the collected date was located out of the range of the predicted value based on the multiplier method, it was considered as the deviation of prediction (DOP). The associations of patient characteristics with DOP were assessed with the use of univariate logistic regression.

**Results:**

Only one article was evaluated as moderate evidence; the remaining articles were evaluated as poor quality. The rate of DOP was 31.82%. In the detailed individual data of included studies, the rate of DOP was 55.44%.

**Conclusion:**

The multiplier method is not reliable in predicting the timing for temporary hemiepiphysiodesis, even though it is prone to be more reliable for the younger patients with idiopathic genu coronal deformity.

## Background

Coronal angular deformity at the metaphyseal level is mainly attributed to three types: congenital, developmental, and acquired [1]. The options for surgical treatment of this condition include guided growth and corrective osteotomy. Osteotomy is often employed for the severe angular deformity or when the physis is closed. However, this procedure has an unpredictable outcome and a high incidence of complications, e.g., compartmental syndrome, neurovascular injury, deep and superficial infections, non-unions, and longer period of rehabilitation [2, 3]. As an alternative to osteotomy, temporary hemiepiphysiodesis seems to be technically easy and minimally invasive. Its application was reported more frequently over the last decade [4–6].

The stapling technique was originally introduced by Blount and Clarke [4]. In 1998, Métaizeau [5] described percutaneous epiphysiodesis using transphyseal screws (PETS). More recently, Stevens designed a system with a non-locking plate and screws called an eight-figure plate, which was explained to function by means of a tension-band mechanism [6]. After that, other modified systems (e.g., tubular plate and reconstruction plate) were also based on the tension-band mechanism. Therefore, the eight-figure plate, tubular plate, and reconstruction were defined as a tension-band plate. All of the apparatuses, such as staple, PETS, and tension-band plate, have the ability to arrest the growth of target physis temporarily. After removing the hardware, the potential of growth is restored. Historically, temporary hemiepiphysiodesis was mainly carried out in the condition of “normal physis” before, and the indication was gradually extended to the cases with “abnormal physis” (e.g., rickets, Blount’s disease, or skeletal dysplasia) because of the sound understanding of this technique [7–9]. After surgery, regular follow-up is necessary. However, it is usually difficult for the patient to be followed in due course. Therefore, it is supposed to be of significant value to predict the rate of correction for temporary hemiepiphysiodesis at the time of clinic visit and make a suitable follow-up strategy. This allows us to evaluate the efficacy of the operated protocol and to predict the outcome after the hardware removal. The multiplier method, which was based on the data from the studies of Anderson et al. [10, 11], was introduced by Paley [1] to calculate the rate of angular correction and predict the timing for temporary hemiepiphysiodesis. However, this method has not been verified in terms of clinical outcome measure.

The purposes of this study were to build up the algorithm to predict the angular correction per year (ACPY) at the various corresponding age by means of multiplier method and to verify the reliability based on the data from the published studies.

## Materials and methods

### Criteria for included studies

The clinical studies were included. The paper retracing was limited to the topic of coronal angular deformity with the treatment of temporary hemiepiphysiodesis. The inclusion criteria are as follows: (1) The patient’s age is ≥3. (2) The data of coronal angular deformity were reported without the records of any other type of lower extremity deformity (e.g., fixed flexion deformity). (3) The rate of angular correction was investigated. The rate of angular correction = the angular change of mLDFA (mechanical lateral distal femoral angle), mMPTA (mechanical medial proximal tibial angle), and HKA (hip-knee angle) during the period between the hardware placement and removal. (4) The stapling and/or PETS and/or tension-band plating was employed. (5) The minimum sample size included 10 patients/limbs. (6) There was no limitation on sex. The exclusion criteria are as follows: (1) The patients had the history of any other knee surgery after hemiepiphysiodesis (e.g., osteotomy and external fixator), (2) ricket disease and coronal angular deformity caused by malignant tumor, (3) the failure of implant or recurrence after hemiepiphysiodesis, and (4) the patients had less than 6-month growth potential.

We retraced the literatures published in English, German, Chinese, and Japanese languages between 1975 and June 2015. A comprehensive search was performed to identify all relevant studies in the following electronic databases: Cochrane Central Register of Controlled Trials (CENTRAL) (The Cochrane Library, current issue), PubMed (1966 to present), and EMBASE (1980 to present). Because of the limited resource of clinical investigations, we used a sensitivity-maximizing strategy. The search strategy combined the study design filter for observational studies adapted from the Scottish Intercollegiate Guidelines Network with the usual Cochrane RCT filter, so that all study designs were captured with this searching strategy, which was demonstrated in Table 1. Two reviewers (ZKW and JD) independently assessed potentially eligible studies. To resolve disagreement between the reviewers, the third review author (LZ) was consulted.

**Table 1:** Search strategy

Part I: anatomic region	Part II: problem	Part III: intervention
Knee	Genu varum	Hemiepiphysiodesis
Knee joint	Genu valgum	Growth guided
Genu	Deformity	Eight plate
Lower extremity	Genu varus	Staple
Tibia	Genu valgas	Temporary epiphysiodesis
Femur	Bow leg	Tension-band plate
	Knock knee	Partial growth plate arrest
	Blount disease	Percutaneous transphyseal screws
	Skeletal dysplasia	
	Angular	
	Metabolic bone diseases	

### Data extraction and management

Two authors (JLL and HL) conducted data extraction independently. The authors were not blinded to the information about the journal name, the authors, the authors’ affiliation, or year of publication. The following data were extracted: method of randomization, blinding (patient/practitioner/analyst), outcomes and follow-up interval, demographics of the identified studies (e.g., number of participants/age/gender/baseline scores of the outcome measures), characteristics of the intervention (stapling versus tension-band plating versus PETS, technique used), loss to follow-up for each group, sponsorship of the trial, and if there was a conflict of interest for any of the study authors. A standardized form was used; it was pilot-tested to ensure that all the interested data were included. Outcome measures included the rate of angular correction. And then, all the detailed data such as age, gender, the rate of angular correction, and the etiology of the individual were extracted from papers if possible. All the data were input into an Excel spreadsheet designed for this study. Two independent review authors (ZKW and JD) assessed the risk of bias. If these two authors did not reach an agreement in this regard, the third review author (LZ) was consulted.

The methodological quality was assessed by means of the 27-item scoring checklist developed by Downs and Black [12]. Quality scores above 20 were considered good; 11–20, moderate; and below 11, poor. The agreement between the two reviewers was evaluated with the Spearman correlation coefficient for interrater agreement and intraclass correlation coefficient (ICC). The review authors pilot-tested the risk of bias assessment from some similar articles. The two authors independently rated all the studies, recorded the final scores for each paper, and resolved any controversy by discussion.

The speed for correction of angular deformity was described quantitatively. The multiplier method was introduced to predict the rate of correction in different age groups [1]. The equation is Mε = Lm/Lm − ((*rα*/57)/*κ*). *α*n = (MεLm − Lm) × 57*κ*/*r*/Mε. ACPY = *αε* − *αε* + 1. Lm is the length of the femur or tibia at skeletal maturity, Mε is the current multiplier, *r* is the width of physis, *α* is the magnitude of angular deformity, ε is the current chronologic age, and *κ* is the percentage of physis in the total tibia or femur. The mean of Lm is 47.23 (femur/boy), 37.29 (tibia/boy), 43.63 (femur/girl), and 34.65 (tibia/girl). The average growth percentile relative to the entire bone until skeletal maturity [1] is *κ* = 0.71 (femur) and 0.57 (tibia). In this equation, the ACPY decreased with the widening of physis. Clinically, the range of width of physis was 5–9 cm described by Bowen et al. [13, 14], and the range of ACPY could be calculated based on this data. We drew a curve of the age-ACPY relationship combined with scatter diagram from the collected date. If the value from collected date was located out of the range of the curve, it was considered as the deviation of prediction (DOP).

## Results

**Fig. 1:**
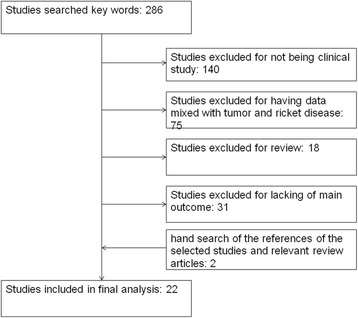
The flowchart of including or excluding review paper studies according to the criteria and their numbers

The initial electronic searches identified 286 citations (Fig. 1). Twenty studies met our inclusion criteria [8, 9, 15–32]. The additional two articles were obtained from hand search [33, 34]. Only one citation was that of RCT [18]. The others were retrospective case series. These studies included a total of 722 patients with 1289 limbs. The detailed data including individual patient extracted from six papers was analyzed independently [9, 17, 28, 32–34].

### Methodological quality

Only one article had its quality score of 19, which was evaluated as moderate [18]. The remaining articles were evaluated as poor quality because they had their scores below 11. The studies which included the records of every individual patient’s detailed data [9, 17, 28, 32–34] were analyzed independently. Spearman’s correlation coefficient for both the interrater agreement and ICC were 0.84 and 0.89, respectively.

### Rate of angular correction

**Fig. 2:**
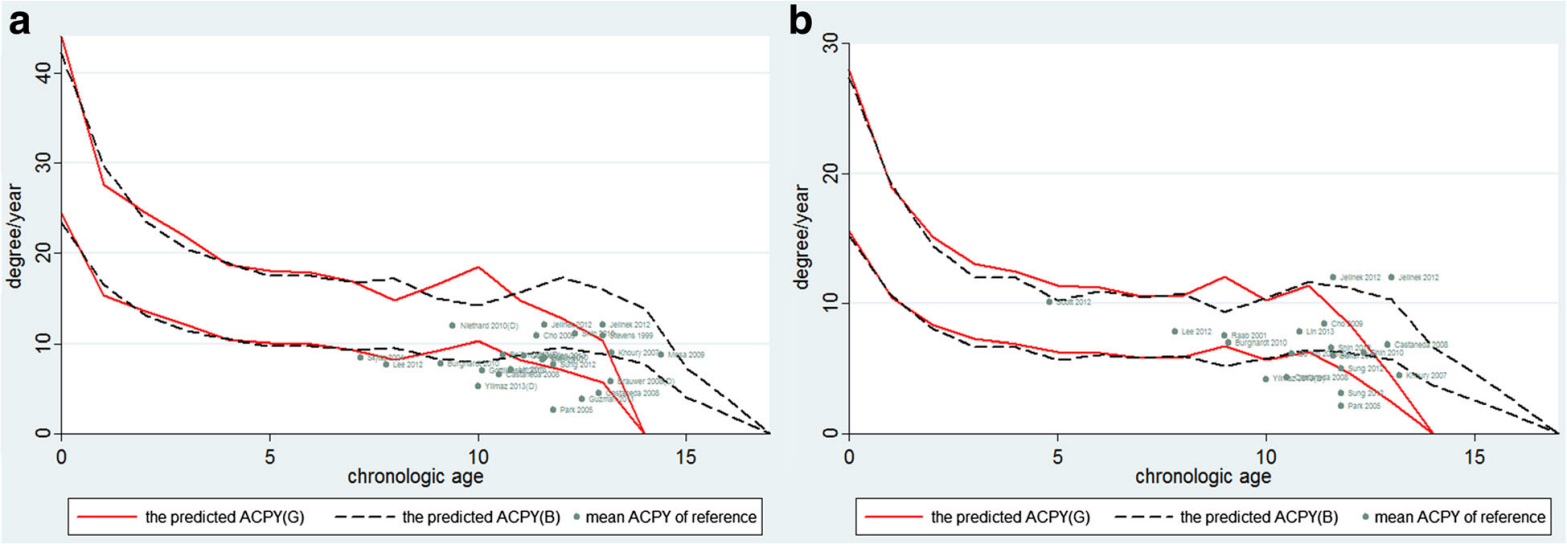
The curve of age-ACPY and scatter diagram of age-ACPM data of included studies. **a** Distal femur. **b** Proximal tibia

**Fig. 3:**
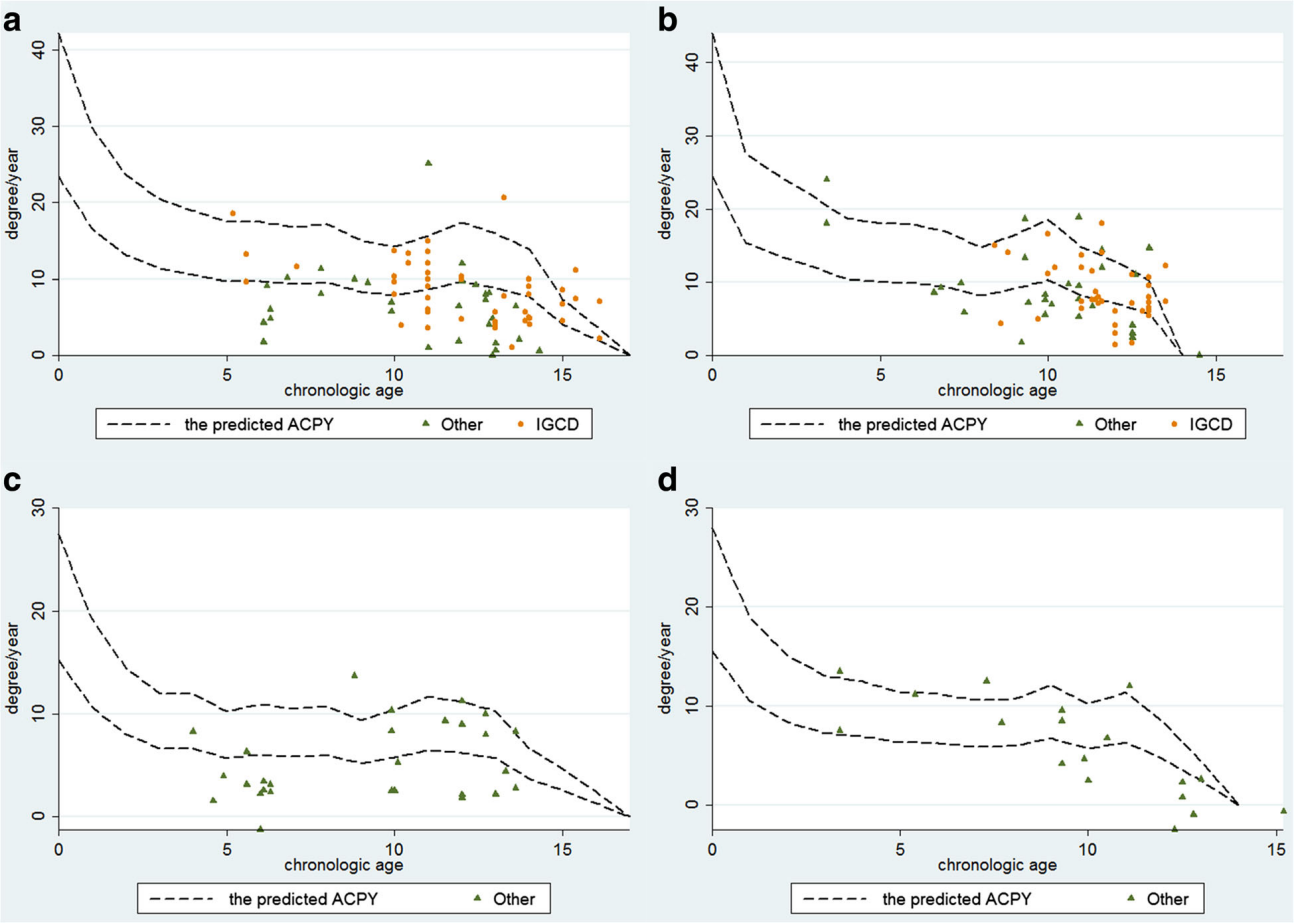
The curve of age-ACPM and scatter diagram of age-ACPM data of each individual from the six papers. **a** Distal femur of boys. **b** Distal femur of girls. **c** Proximal tibia of boys. **d** Proximal tibia of girls. (*IGCD* idiopathic genu coronal deformity. Other: non-IGCD)

The multiplier method was used to estimate the rate of correction [1]. The result was shown in Table 2. Supposing that the range of physeal width was from 5 to 9 cm, according to Table 2, we could calculate the ACPY. In case that a 10-year-old male goes to see a doctor, the predicted ACPY will be from 7.9 to 14.23° with his conditions at the distal femur, while 5.80 to 10.44° with his conditions at the proximal tibia. Bowen et al. [13, 14] reported that 5° of angular correction could be expected for each year of the remaining growth after a tibial hemiepiphysiodesis and 7° after a femoral hemiepiphysiodesis. The result was also similar with the data based on the multiplier method. The mean ACPY in terms of mLDFA and mMPTA ranged from 2.63° to 12° and 2.11° to 12°, respectively, in the included studies. Figure 2 shows that in the femur group, the rate of DOP was 10/25 and in the tibia group, it was 4/19. In the detailed individual data of the included studies, 9.9% cases had the value of ACPY located above the range of curve while 45.54% cases had the value located underneath the range of curve (Fig. 3).

**Table 2:** The predicted ACPY (degree/year) based on the multiplier method

Age (B)	ACPY (F)	ACPY (T)
0	23.42–42.16	15.23–27.41
1	16.54–29.77	10.65–19.17
2	13.13–23.63	8.05–14.50
3	11.38–20.48	6.67–12.01
4	10.50–18.90	6.66–11.98
5	9.72–17.50	5.69–10.24
6	9.72–17.50	6.09–10.96
7	9.32–16.78	5.83–10.50
8	9.56–17.20	5.97–10.75
9	8.35–15.03	5.21–9.38
10	7.90–14.23	5.80–10.44
11	8.71–15.68	6.50–11.69
12	9.64–17.36	6.22–11.19
13	8.86–15.95	5.72–10.30
14	7.71–13.87	3.70–6.66
15	4.08–7.35	2.59–4.66
16	2.10–3.78	1.33–2.40
Age (G)	ACPY (F)	ACPY (T)
0	24.45–44.01	15.56–28.00
1	15.36–27.64	10.50–18.90
2	13.61–24.51	8.38–15.09
3	12.09–21.77	7.26–13.07
4	10.39–18.70	6.92–12.46
5	10.04–18.08	6.32–11.38
6	9.93–17.88	6.25–11.25
7	9.35–16.83	5.88–10.58
8	8.20–14.75	5.93–10.67
9	9.16–16.49	6.73–12.11
10	10.30–18.55	5.68–10.22
11	8.19–14.73	6.32–11.38
12	7.12–12.82	4.63–8.33
13	5.71–10.29	2.45–4.41

*F* operative part in distal femur, *T* operative part in proximal tibia, *B* boys, *G* girls, *ACPY* angular correction per year

## Discussions

Temporary hemiepiphysiodesis allows progressive correction of the deformity. In children with open physis, angular deformity can be corrected by asymmetrically suppressing the physis and taking advantage of the physeal growth. In view of the close relationship between the patients’ age and rate of correction, a predicted chart is necessary to help surgeons decide the timing of operation and make a suitable follow-up strategy. In history, based on the data from Anderson et al. [10, 11], Bowen and Paley developed different strategies to predict the proper time to perform the hemiepiphysiodesis, respectively [1, 13, 14]. Bowen’s chart was designed for permanent hemiepiphysiodesis, while Paley’s method was more suitable for temporary hemiepiphysiodesis [1, 13, 14]. Paley’s method had been considered as a reasonable method to estimate the ultimate limb length at maturity by some authors [35, 36]; nevertheless, others questioned the reliability and accuracy of this method [37–40]. The debate was focused on whether skeletal age could be replaced with chronologic age to predict the potential of growth. To our knowledge, no study was reported to examine the reliability of this method in predicting the potential of angular deformity. It is necessary to verify the reliability of this method.

According to Fig. 2, we have to suspect the reliability of the multiplier method in predicting the ACPY.

First, according to Anderson’s data, the annual increment of growth was stable before puberty, while the rate of growth became more rapid once the growth spurt started [10]. It was supposed that it was reliable to predict the potential of growth in childhood by means of chronologic age. After onset of puberty, skeletal age, as a more objective criterion of relative maturity, is preferable. Other authors raised a similar idea [36, 41, 42]. In our article, based on the transformation of multiplier method, the range of angular correction per year could be calculated. Figure 2 shows that the curve of ACPY was flat over a long period of time (from age 5 to age 10), then it became fluctuant after age 10. Our finding was similar with that by Anderson and other authors. The age of 10 years was set up as a transition point in our paper, according to the beginning of the early adolescent growth spurt. The rate of DOP in the younger patients (≤10) was significantly lower than that in the older patients. In the paper by Brauwer and Moens [34], the chronologic age and skeletal age of some patients were recorded. When the value of the chronologic age was replaced with the skeletal age in the multiplier method, the number of DOP was significantly decreased (15 to 6). Lee et al. [38] supposed that whichever age was used, the multiplier method was statistically inaccurate in predicting ultimate limb length at maturity. However, this research included some patients with “abnormal physis,” which was a risk factor of the DOP mentioned below. Skeletal age might be more accurate in predicting the angular correction than chronologic age after onset of puberty. Above all, the power of this study was not strong enough to verify its own opinion because of the smaller size of the samples.

Second, the etiology of coronal angular deformity was different. The potential of growth was different between the IGCD and non-IGCD. Boero et al. [43] and Wiemann et al. [44] reported that the speed of correction was faster in idiopathic deformity than in pathological deformity, Castañeda thought that the rate of correction of “abnormal physis” was unpredictable [19], and Park et al. [29] and Westberry et al. [45] also performed similar opinion. Oto et al. [46] even suggested that it was useless to treat severe adolescent Blount disease with TH due to the low rate of correction. The etiology included in this paper presents a number of causes such as IGCD, Blount’ disease, congenital femoral deficiency, fibular hemimelia, metaphyseal dysplasia, multiple epiphyseal dysplasia, Morquio syndrome, pseudoachondroplasia, enchondral dysostosis, post-traumatic deformity, Ellis-van Creveld syndrome, multiple exostoses, clubfoot, and neurofibromatosis. Figure 3 shows that comparing with IGCD, the ACPY value from most patients with non-IGCD was located under the range of curve. It is supposed that the potential of growth in the abnormal physis group was lower than that in the normal physis group. It might be the reason why the rate of DOP was high. For patients with “abnormal physis,” the method was also unreliable.

It was the shortcomings of this review that only one RCT study was included while the other papers were admitted into the category of low-level evidence. All of these parameters and outcome measures were objective; therefore, the authors of this systemic study evaluate that the outcome may not likely be influenced by the lack of blinding. The actual evidence of this article might be upgraded. Six papers were combined and analyzed by univariate logistic regression. Due to the limit of sample size, only the femur was analyzed. Despite that strict inclusion and exclusion criteria were employed to overcome the difference of baseline characteristics, some risk factors (e.g., race, operative procedure, obesity, and severity of deformity) might lead to confounding. McIntosh et al. [47] suggested that these were risk factors for implant failure. Park et al. [29] thought that these factors would reduce the potential of angular correction. However, the sample size was not large enough to provide sufficient strength. In most of the included studies, there were no records as to BMI and preliminary angle of deformity in every individual patient before the treatment; therefore, these factors were not included into the analysis in our study. RCT study is required to analyze these confounding factors in the future.

More attention should be paid to the width of physis which supposedly influences the rate of correction. The more the width of physis is, the less ACPY is for angular correction according to the multiplier method or the Bowen chart [10, 12, 42]. However, it was rarely emphasized in all the included studies. It still needs to be verified in further clinical studies.

## Conclusion

In conclusion, based on the analysis of data from the published studies included in this systemic review, the multiplier method is not reliable in predicting the rate of correction, even though it is prone to be more reliable for younger patients with idiopathic genu coronal deformity. The age older than 10 and the condition of non-idiopathic genu coronal deformity may be the risk factors for deviation of prediction.

## Abbreviations

ACPY: Angular correction per year
DOP: Deviation of prediction
